# Effects of Apelin on Left Ventricular-Arterial Coupling and Mechanical Efficiency in Rats with Ischemic Heart Failure

**DOI:** 10.1155/2019/4823156

**Published:** 2019-06-17

**Authors:** Qiufang Ouyang, Tao You, Jinjian Guo, Rong Xu, Quehui Guo, Jiqin Lin, Hongjia Zhao

**Affiliations:** ^1^Department of Ultrasonography, Second Affiliated People's Hospital of Fujian Traditional Chinese Medicine University, Fuzhou, Fujian, China; ^2^Department of Cardiology, Second Affiliated People's Hospital of Fujian Traditional Chinese Medicine University, Fuzhou, Fujian, China; ^3^Department of Cardiology, First Affiliated People's Hospital of Fujian Traditional Chinese Medicine University, Fuzhou, Fujian, China

## Abstract

Apelin plays important roles in cardiovascular homeostasis. However, its effects on the mechanoenergetics of heart failure (HF) are unavailable. We attempted to investigate the effects of apelin on the left ventricular-arterial coupling (VAC) and mechanical efficiency in rats with HF. HF was induced in rats by the ligation of the left coronary artery. The ischemic HF rats were treated with apelin or saline for 12 weeks. The sham-operated animals served as the control. The left ventricular (LV) afterload and the systolic and diastolic functions, as well as the mechanoenergetic indices were estimated from the pressure-volume loops. Myocardial fibrosis by Masson's trichrome staining, myocardial apoptosis by TUNEL, and collagen content in the aorta as well as media area in the aorta and the mesenteric arteries were determined. Our data indicated that HF rats manifested an increased arterial load (Ea), a declined systolic function (reduced ejection fraction, +dP/dt_max_, end-systolic elastance, and stroke work), an abnormal diastolic function (elevated end-diastolic pressure, *τ*, and declined −dP/dt_max_), and decreased mechanical efficiency. Apelin treatment improved those indices. Concomitantly, increased fibrosis in the LV myocardium and the aorta and enhanced apoptosis in the LV were partially restored by apelin treatment. A declined wall-to-lumen ratio in the mesenteric arteries of the untreated HF rats was further reduced in the apelin-treated group. We concluded that the rats with ischemic HF were characterized by deteriorated LV mechanoenergetics. Apelin improved mechanical efficiency, at least in part, due to the inhibiting cardiac fibrosis and apoptosis in the LV myocardium, reducing collagen deposition in the aorta and dilating the resistant artery.

## 1. Introduction

The interaction between the left ventricle (LV) and the arterial system, usually termed ventricular-arterial coupling (VAC), is recognized nowadays as a key determinant of global cardiovascular performance [1]. The cardiovascular system is structured to provide adequate pressure and flow to the tissues. Aortic elastic properties and total arterial compliance are important determinants of the left ventricular function and coronary blood flow. Studying the LV efficiency requires investigating not only the performance of the LV itself but also the properties of the arterial system.

Heart failure (HF) following myocardial infarction (MI) is associated with cardiac and vascular alterations. The cardiac remodeling is characterized by LV dilation and pump dysfunction, indicating decreased LV end-systolicelastance (Ees) and stroke work (SW). Meanwhile, the vascular changes are manifested as the compliance decreased, which led to an increase in effective arterial elastance (Ea). Accordingly, VAC and mechanical efficiency in the LV were altered during ischemic HF [30].

Apelin is the endogenous ligand of the G protein-coupled receptor APJ. Now, a second ligand for the apelin receptor has been discovered in the fish Danio, called Elabela [2] or Toddler [3]. Elabela/Toddler has also been shown to be present in humans and act as the apelin receptor and is downregulated like apelin in cardiovascular diseases. The Toddler/apelin/APJ system plays important roles in adjusting the blood pressure, the pulmonary artery pressure, and the cardiac function [4–6]. Compelling evidence indicated that the exogenous apelin treatment significantly increased the LV stroke volume [7], enhanced the cardiac contractility [8], reduced the ventricular preload and afterload [9], or caused vasodilation [10] in various *in vivo* models. However, the effect of apelin on the interaction of LV and the arterial system in HF rats has not been reported yet.

Therefore, the aim of this work was to investigate the effects of apelin on VAC and mechanical efficiency with pressure-volume (P-V) analysis in a model of ischemic HF. Then, the morphological changes of LV and the artery were investigated, trying to elucidate the mechanism underlying these effects from the perspective of histomorphology.

## 2. Materials and Methods

### 2.1. Animal Experiment Protocol

All of the procedures and protocols were approved by the Animal Care Committee of Fujian Traditional Chinese Medicine University and followed the guidelines of the Animal Management Rules of the Chinese Ministry of Health.

The heart failure model was established in 10-week-old male Wistar rats (Shanghai Laboratory Animal Center, Chinese Academy Sciences) by left anterior descending (LAD) ligation, as described previously [11]. Briefly, rats were anesthetized with a combination of 80 mg/kg ketamine and 5 mg/kg acepromazine (both form Sigma-Aldrich, St. Louis, MO, USA), and the left thoracotomy was performed. The heart was exteriorized, and the LAD was ligated 2 mm from its origin with a Prolene 6-0 suture. In the sham-operated animals, the suture was passed but not tied. After the procedure, the animals were closed in 3 layers. Immediately after surgery, the left ventricular dimensions, the ejection fraction (EF), and the MI size were assessed by echocardiography. Following echocardiography, the rats with similar MI size (both average in size and variance) were divided into two groups: the apelin group (*n* = 20) received (Pyr1)-apelin-13 (GL Biochem Ltd., Shanghai, China), 200 *μ*g/kg/day intraperitoneal injection, initiated after echocardiography, once a day for 12 weeks; and the control group (*n* = 20) as well as the sham-operated rats (*n* = 15), treated with isovolume saline. At the end of the treatment, the rats were weighed and the hemodynamics was analyzed. Then, these animals were killed for morphologic study.

### 2.2. Assessment of the Hemodynamics and the Left Ventricular Function by Pressure-Volume Analysis

12 weeks after the treatment, the cardiac hemodynamic parameters were determined. For this, the rats were anesthetized with the method as mentioned above and ventilated. A small incision was made to the right of the midline in the neck. A polyethylene catheter was inserted into the left external jugular vein for fluid administration. A 2-Fr microtip pressure-conductance catheter (SPR-838, Millar Instruments, Houston, TX) was inserted into the right carotid artery and advanced into the ascending aorta. After stabilization for 5 min, the mean arterial pressure (MAP) and the heart rate (HR) were recorded by the PowerLab data acquisition system (ADInstruments, Australia). Then, the catheter was advanced into the LV under pressure control. With the use of a special pressure-volume (P-V) analysis program, the traditional load-dependent hemodynamic indices, such as LV end-systolic pressure (ESP), LV end-diastolic pressure (EDP), the maximal slopes of LV systolic pressure increment (+dP/dt_max_) and diastolic pressure decrement (-dP/dt_max_), and the isovolumic relaxation time constant (*τ*) were determined. The slope of the LV end-diastolic PV relationship (EDPVR) was calculated as a reliable indicator of LV stiffness [12]. Meanwhile, the load-independent indices, i.e., the end-systolic elastance (Ees), the arterial elastance (Ea, calculated as ESP/SV), the stroke work (SW), and the P-V area (PVA, the specific area in the P-V plane bounded by the end-systolic and end-diastolic P-V relationship lines and the systolic segment of the P-V loop), were calculated by transient occlusion of the inferior caval vein. Then, the VAC ratio (as Ea/Ees) and the mechanical efficiency (as SW/PVA) were calculated to assess the LV mechanoenergetics [13].

### 2.3. Determination of Myocardial Fibrosis with Masson Staining and Apoptosis with Terminal dUTP Nick End Labeling Assay

The hearts of the rats in each group were harvested, fixed in 4% paraformaldehyde, embedded in paraffin, and cut into 5 *μ*m sections. Subsequently, the LV myocardial sections from the midpapillary muscle level were subjected to hematoxylin and eosin, Masson's trichrome, and terminal dUTP nick end labeling (TUNEL) staining. MI size was expressed as an average percentage of the LV endocardial and epicardial circumferences that was identified as infarct in the Masson's trichrome staining sections. Myocardial fibrosis was expressed as a percentage of fibrotic area to the left ventricular area (% of LV) in an average of 5 sections in each heart. The number of TUNEL-positive cardiomyocyte nuclei was counted manually in whole, noninfarcted myocardium, including the LV posterior wall and septum, under light microscopy from each LV short-axis section. Only the nuclei clearly located within cardiomyocytes were counted and expressed as a percentage of total myocytes in a given LV section.

### 2.4. Histology and Morphometric Analysis of the Aorta and the Mesenteric Artery

The thoracic aorta and mesenteric artery specimens were collected and stained with hematoxylin and eosin staining. The media cross-sectional area (CSA, defined as the area between the internal and external elastic laminae) and the media-to-lumen ratio (defined as the ratio of the medial area and the lumen area times 100%) in the aorta and the mesenteric artery were determined. Meanwhile, the collagen deposition in the aorta was determined by Masson's trichrome staining. The collagen content was represented by collagen area/media area in order to normalize the vessels with different sizes. Photos were taken by a microscope (BX52, Olympus, Tokyo, Japan), and the data were analyzed by the Image-Pro Plus 5.0 analysis software (Media Cybernetics, USA).

### 2.5. Statistical Analysis

All the values were expressed as the mean ± standard deviation unless otherwise indicated. The group comparisons were performed with one-way ANOVAs, followed by a Bonferroni test. The Mann-Whitney *U* test was used if the variance was not normally distributed significantly. The statistical analysis was performed using the SPSS 17.0 software. A value of *P* < 0.05 was considered statistically significant.

## 3. Results

### 3.1. Apelin-13 Improved the Left Ventricular Dysfunction in Rats with Ischemic Heart Failure

There was no mortality in the sham-operated group and apelin-treated HF group. In the untreated HF group, two rats were excluded from further study due to a myocardial infarction size less than 20%. And four rats died 24 hours after coronary ligation surgery. Additionally, two rats died during the assessment of hemodynamics. And so, only 12 animals remained alive in the untreated HF group at the study termination. Therefore, 12 rats were eventually included in each group for the following experiments.

**Figure 1:**
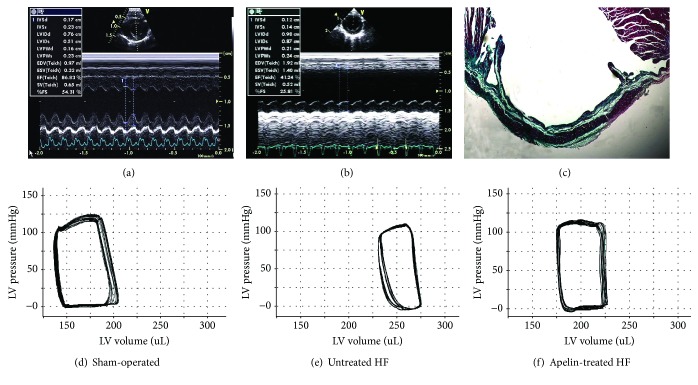
Representative short-axis echocardiographic images at midpapillary muscle level before treatment, gross left ventricular morphology, and pressure-volume loops at the study termination.

In rats following LAD ligation, the LV dimensions enlarged, the EF dropped, the motion of the anterior wall attenuated, and the systolic thickening rate of the anterior wall disappeared, indicating a successful ischemic HF model (Figure 1(b)). Obvious fibrosis in the myocardial infarction region was observed in the untreated HF rats at the study termination as indicated by Masson's trichrome staining (Figure 1(c)). In comparison with the sham-operated rat (Figure 1(d)), the rightward shift of the end-systolic pressure-volume relationship and the narrowed P-V loop (Figure 1(e)) in the HF animals were partially reversed by apelin treatment (Figure 1(f)).

LVSP in the untreated HF was significantly lower than that in the sham-operated group (105.56 ± 12.49 mmHg vs 136.81 ± 9.06 mmHg). Apelin treatment induced a further significant reduction of LVSP by 11.7% compared to the untreated HF animals. The heart rate differed insignificantly among the three groups (Table 1). Increased vascular afterload parameters (Ea) in the untreated HF group was partially restored by apelin treatment (0.51 ± 0.04 mmHg/*μ*l in the sham, 0.74 ± 0.08 mmHg/*μ*l in the untreated, and 0.46 ± 0.05 mmHg/*μ*l in the apelin group).

EF, +dP/dt_max_, Ees, and SW were lower in the untreated HF group than those in the sham-operated group, indicating a significant decline of the LV systolic function. Apelin treatment increased those indices by 27.4% for EF, 18.4% for +dP/dt_max_, 57.5% for Ees, and 64.5% for SW.

Meanwhile, as compared to the sham-operated rats, an impaired diastolic function was observed in the untreated HF group as evidenced by the elevated EDP and the slopes of the EDPVR and *τ* as well as by the reduced -dP/dt_min_. Apelin treatment improved the diastolic function as evidenced by reduced EDP (16.19 ± 5.92 mmHg vs 25.65 ± 4.93 mmHg) and the slopes of the end-diastolic pressure-volume relationship (0.044 ± 0.003 mmHg/*μ*l vs 0.056 ± 0.008 mmHg*μ*l) and *τ* (10.42 ± 0.78 msec vs 13.91 ± 1.78 msec) whereas elevated -dP/dt_min_ (6746.29 ± 714.83 mmHg/s vs 5150.77 ± 629.05 mmHg/s) when compared to the untreated HF group.

In comparison with the sham-operated animals, Ea/Ees rose markedly in the untreated HF group, suggesting a deterioration of VAC (1.62 ± 0.13 vs 0.52 ± 0.08). Concurrently, SW/PVA dropped obviously due to decreased SW and slightly elevated PVA. As a consequence, mechanical efficiency remained significantly impaired at 0.34 ± 0.04, as compared with the sham-operated group (0.56 ± 0.08). Following the treatment with apelin-13, VAC and mechanical efficiency were significantly improved by 42.6% and 41.2%, respectively, as compared to the untreated HF rats.

### 3.2. Apelin-13 Improved the Structural Disorder and Myocardial Fibrosis in Rats with Ischemic Heart Failure

**Figure 2:**
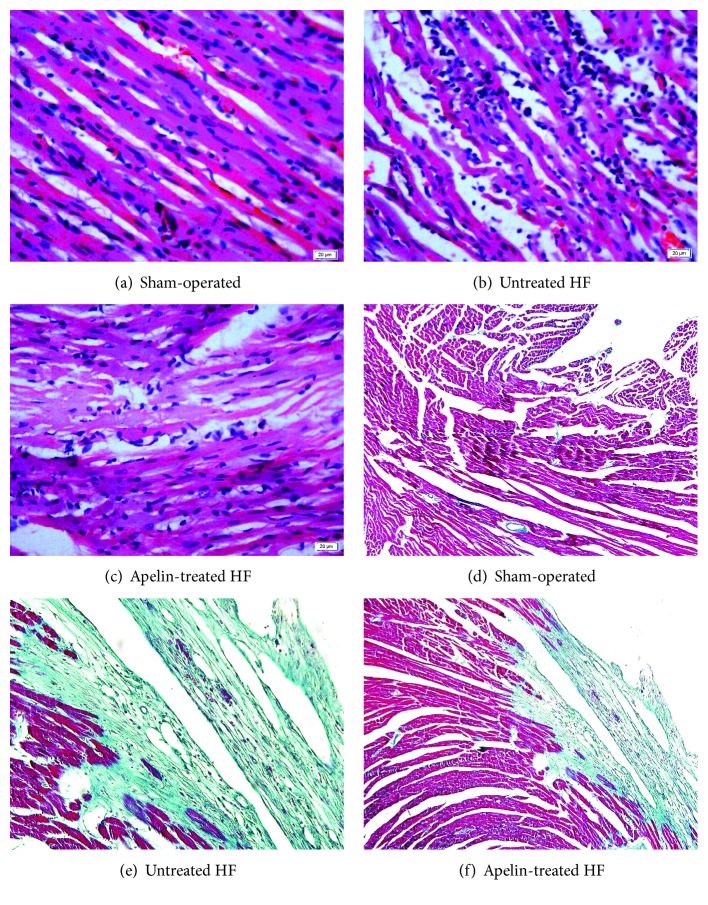
The morphological changes as illustrated by hematoxylin and eosin staining and Masson's trichrome staining. For Masson's trichrome staining, fibrosis was stained blue, whereas cytoplasm red. Untreated HF animals displayed disorder structure and fibrotic myocardium, which were ameliorated by apelin treatment.

Hematoxylin and eosin staining was used to evaluate the morphological changes. The left ventricle in the sham-operated group (Figure 2(a)) exhibited normal cardiomyocyte structure, with a clear texture and vein, and plump cytoplasm (less gaps and spaces between the myofilaments). However, myocardium in the untreated HF group (Figure 2(b)) showed disordered structure. The myofilaments were rougher with wave-like changes and the boundary of textures and veins was unclear. While in the apelin-treated group (Figure 2(c)), the above histopathological changes were ameliorated as compared with the untreated group.

Cardiac fibrosis was evaluated by Masson's trichrome staining. The LV myocardium in the untreated HF group (Figure 2(e)) exhibited numerous collagen fiber (blue) compared with the sham-operated group (Figure 2(d)). However, following the treatment with apelin-13 (Figure 2(f)), the percentage of fibrosis was significantly attenuated by 31.1% compared with the untreated HF group (Table 1).

### 3.3. Apelin-13 Inhibited Cardiac Cell Apoptosis in Rats with Ischemic Heart Failure

**Figure 3:**
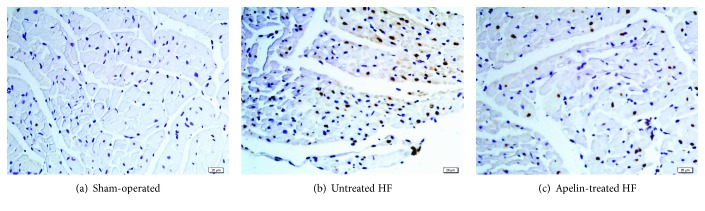
Representative TUNEL staining slices for left ventricular myocardium. TUNEL: terminal dUTP nick end labeling. Apoptotic nuclei were stained brown. Apelin alleviated myocardial apoptosis induced by coronary artery ligation.

Representative slices of myocardium in the LV posterior wall of TUNEL staining (Figure 3) and the average number of positively stained nuclei for each group were shown (Table 1). After 12 weeks of treatment, the number of TUNEL-positive nuclei in the apelin-treated group (316 ± 46 per 10^5^ myocytes) was significantly lower than that in the model group (629 ± 91 per 10^5^ myocytes). And sparse apoptotic nuclei were observed in the sham-operated group.

### 3.4. Effect of Apelin on Morphological Characteristics of the Aorta and the Mesenteric Arteries

**Figure 4:**
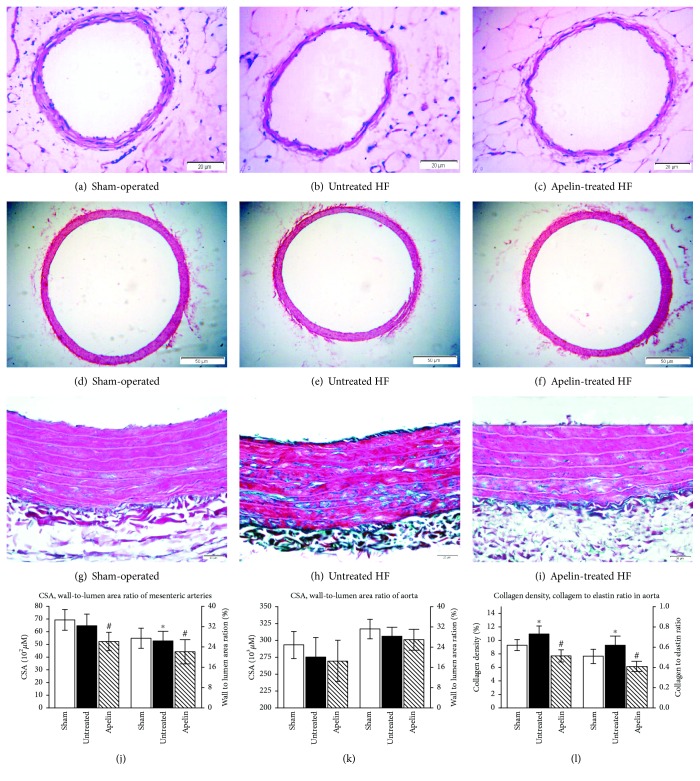
The media cross-sectional area in thoracic and mesenteric arteries depicted by hematoxylin and eosin staining and the collagen density in thoracic aorta indicated by Masson's trichrome staining. Representative transverse sections of the complete mesenteric arteries (a, b, and c) and aortas (d, e, and f) stained with hematoxylin and eosin. Aortic collagen deposition was shown by Masson's trichrome staining. Collagen was stained green while muscle and cytoplasm were red (g, h, and i). Quantitative analysis of media cross-sectional area in the mesenteric and thoracic arteries (j, k) and collagen in the aortas (l). CSA: media cross-sectional area. Apelin reduced media CSA and wall-to-lumen ratio in the mesenteric arteries and alleviated aorta collagen accumulation, but it did not affect media CSA and wall-to-lumen ratio in the aortas.

Compared with the sham-operated animals (Figure 4(a)), media thickness reduced significantly and lumen diameter augmented slightly in the mesenteric arteries from rats of the untreated HF group (Figure 4(b)). Those resulted in a smaller media­to­lumen ratio in untreated HF rats. There was no significant difference in CSA between the untreated HF and sham-operated groups. Apelin treatment attenuated CSA by 19.2% and media­to­lumen ratio by 16.3% when compared with the untreated HF animals (Figures 4(c) and 4(j)).

In the thoracic aorta, no statistically significant changes of the layers of the elastic lamina, the media CSA, the lumen diameter, the media thickness, or the wall-to-lumen ratio were observed among the groups (Figures 4(d), 4(e), 4(f), and 4(k)). However, higher collagen deposition was present in the untreated HF group (Figure 4(h)) compared with sham-operated rats (Figure 4(g)) (9.35%±0.81% vs 10.91%±1.25%) as demonstrated by Masson's trichrome staining. Apelin alleviated aorta collagen density without a change in the elastin area (data not shown), leading to a declined collagen/elastin ratio in the aorta (Figure 4(i)).

## 4. Discussion

This study provides evidence that deteriorated LV mechanoenergetics (mechanical efficiency and ventricular-arterial coupling) in HF rats can be improved by apelin treatment via elevating Ees and SW, decreasing Ea, and unaltering PVA. And this effect is, at least in part, due to the inhibiting cardiac fibrosis and apoptosis in LV myocardium, reducing collagen deposition in the aorta and dilating the resistant artery.

### 4.1. Apelin Modified the Arterial Properties in Rats with Ischemic Heart Failure

The aorta functions not only as a conduit delivering blood to tissues but also as an important modulator of the entire cardiovascular system, buffering the intermittent pulsatile output from the heart to provide steady flow to the capillary beds. The arterial compliance is an important determinant of the left ventricular function and coronary blood flow. Arterial system stiffness can be characterized by Ea, an integrative index that incorporates the principal elements of arterial load, including peripheral vascular resistance, total arterial compliance, and characteristic impedance [14]. Accordingly, the elevated Ea can result from either peripheral vasoconstriction or decrease in arterial compliance or a combination of both. Elastic and collagen fibers and their normal arrangement primarily determine the arterial compliance. The increase of collagen/elastin ratio represented the decline in arterial compliance. In this study, our data showed that increased Ea in the untreated HF rats was reversed by apelin treatment. In parallel, our result suggested that higher collagen deposition in the aortas was present in the untreated HF group, which can be alleviated by apelin treatment. And our result was partially substantiated by the observation that collagen area increased by 59% in the carotid artery of MI rats at 3 weeks of post-LAD ligation [15]. This was somewhat at odds with the report that media collagen density was not different between the sham-operated and untreated HF animals, either in the abdominal aorta, the femoral, or the mesenteric arteries at 90 days following LAD ligation [16]. The inconsistency may be partly explained by the difference in the disease course of HF or the type of artery studied.

There are also conflicting results on morphologic changes of mesenteric arteries in rats with ischemic HF. Several studies show that no morphologic changes were seen at 3 and 5 weeks of postoperation [17, 18], whereas others demonstrated an increased lumen diameter and reduced media thickness in the mesenteric arteries at 12 weeks of postinfarct [19]. Our results confirm the latter finding that the media­to­lumen ratio of the mesenteric arteries was lower in the untreated HF than that in the sham-operated animals. Additionally, our data indicated that apelin attenuated CSA and media­to­lumen ratio in the mesenteric arteries in the HF animals. To the best of our knowledge, no investigation has elaborated the effect of apelin on the remodeling of the mesenteric arteries in HF rats as yet.

In brief, apelin may reduce Ea by inhibiting the collagen deposition in the aorta and by dilating the resistance vessels. Presumably, the molecular mechanism may be related to the activation of nitric oxide (NO) pathways [20].

### 4.2. Apelin Improved LV Structural and Functional Characteristics

EF, +dP/dt_max_, and SW were elevated in the apelin-treated rats, suggesting an increased LV systolic performance. Myocardial contractile dysfunction in heart failure is characterized by a decrease in contraction and prolonged relaxation. These alterations are mainly due to the changes in intracellular Ca^2+^ transients (CaT), Ca^2+^ sensitivity of the contractile elements, and/or contractile proteins [21]. Although the molecular mechanisms underlying its inotropic effect are not clarified in this investigation, activation of protein kinase C*ε* (PKC*ε*) and extracellular signal-regulated kinase (ERK1/2) signaling might be involved [22].

Additionally, apoptosis is also responsible for the decline of pump function. The present data indicated that the numbers of TUNEL-positive nuclei in the apelin group were significantly lower compared to the untreated HF group. This observation is supported by the report that apelin-13 exerts antiapoptotic effects in the rats with myocardial infarction [23]. The possible mechanisms are related to the phosphatidylinositol-3-kinase (PI3K)/Akt, ERK1/2, caspase signaling, and autophagy pathways [24].

The impaired active relaxation and the increased myocardial stiffness are both responsible for the diastolic dysfunction. LV active relaxation is an active, energy-consuming process and depends mostly on calcium uptake by the sarcoplasmic reticulum during diastole [25], while the main determinant of myocardial stiffness is the collagen accumulation in the extracellular matrix [26]. In our investigation, EDP, slope of EDPVR, *τ*, and -dP/dt_max_ were partially reversed by apelin treatment, suggesting improved active relaxation and end-diastolic stiffness. This observation was precisely reflected by our investigation, since the slope of EDPVR (the index of myocardial stiffness), along with the Masson's score, significantly increased in the HF model group and regressed by apelin treatment.

### 4.3. Apelin Ameliorated Mechanoenergetic Indices of Ventricular-Arterial Coupling and Mechanical Efficiency

Imbalance between energy production and consumption was observed in heart failure [27]. In this investigation, mechanoenergetic changes were determined by the ratios of Ea/Ees and SW/PVA via P-V analysis [28]. Ahmet et al. [29] reported that the rats with ischemic heart failure manifested mismatched VAC due to increased Ea and decreased Ees. Another study corroborated this finding that VAC and mechanical efficiency were severely deteriorated in pigs with myocardial infarct [13]. Similarly, our investigation indicated that LAD ligation induced inefficient VAC. In addition, our data demonstrated that apelin improved VAC, suggesting its optimal efficiency of blood transfer from the heart to the arterial system.

SW is the external mechanical work performed by the LV during a single heart cycle. PVA represents the total mechanical energy generated by ventricular contraction. PVA is an index of LV total energy expenditure mechanical energy and linearly related to total myocardial oxygen consumption [30]. We found that SW was enhanced while PVA was altered insignificantly by apelin treatment, indicating that apelin increased cardiac contractility with no significant change of metabolic needs of viable myocardium. Thus, apelin is a potential innovative treatment for heart failure.

Most studies hitherto have focused on the effects of apelin treatment on the single aspect of the heart or the vessels. And there is a paucity of information on the effects of apelin on the interrelationship between these two aspects. Our study suggested myocardial infarction resulted in structural and functional alterations at both the arterial level and the cardiac level, which is valuable for the global understanding of the pathophysiological phenomena in the development of HF. Furthermore, our result provided novel morphological evidences concerning the cardiovascular protective potential of apelin on heart failure. Treatment of HF should therefore not only be directed to increase LV contractility but also to reduce arterial elastance.

Further research at the endothelial function, renin-angiotensin system, or intracellular Ca^2+^ may facilitate our understanding of the effects of apelin on mechanoenergetics in rats with HF. Additionally, caution is needed in translating these results into clinical practice.

## 5. Conclusions

LV performance and arterial properties are impaired in tandem in rats with ischemic heart failure, which resulted in ventricular-arterial mismatch and mechanical efficiency deterioration. Apelin improved mechanoenergetics, at least in part, due to its inhibiting cardiac fibrosis and apoptosis in LV myocardium, reducing collagen deposition in the aorta and dilating the resistant artery.

## Figures and Tables

**Table 1:** Hemodynamic and histological measurements among the groups.

	Sham-operated (*n* = 12)	Untreated HF (*n* = 12)	Apelin-treated HF (*n* = 12)
Final body weight (g)	542.58 ± 38.29	525.16 ± 24.45	538.65 ± 29.48
Hemodynamics			
LVSP (mmHg)	136.81 ± 9.06	105.56 ± 12.49^∗^	93.28 ± 11.64^#^
Ea (mmHg/*μ*l)	0.51 ± 0.04	0.74 ± 0.08^∗^	0.46 ± 0.05^#^
Ejection fraction (%)	68.64 ± 4.55	43.13 ± 6.09^∗^	54.96 ± 5.24^#^
+dP/dt_max_ (mmHg/s)	8514.12 ± 745.83	5950.74 ± 729.60^∗^	7047.67 ± 635.58^#^
Ees (mmHg/*μ*l)	1.06 ± 0.25	0.40 ± 0.08^∗^	0.63 ± 0.15^#^
SW (mmHg/ml)	9.16 ± 10.24	6.02 ± 1.18^∗^	7.35 ± 0.93^#^
−dP/dt_max_ (mmHg/s)	8129.41 ± 802.95	5150.77 ± 629.05^∗^	6746.29 ± 714.83^#^
EDP (mmHg)	5.84 ± 1.47	25.65 ± 4.93^∗^	16.19 ± 5.92^#^
Slope of EDPVR (mmHg/*μ*l)	0.035 ± 0.003	0.056 ± 0.008^∗^	0.044 ± 0.003^#^
*τ* (msec)	9.14 ± 0.49	13.91 ± 1.78^∗^	10.42 ± 0.78^#^
Ea/Ees	0.52 ± 0.08	1.62 ± 0.13^∗^	0.93 ± 0.05^#^
Pressure-volume area (mmHg/ml)	16.44 ± 1.18	17.70 ± 10.24	16.62 ± 0.93
Heart rate (bpm)	405.43 ± 20.41	412.15 ± 54.26	378.26 ± 56.64
SW/PVA	0.56 ± 0.08	0.34 ± 0.04^∗^	0.48 ± 0.05^#^
Histology			
Collagen density in LV (%)	1.92 ± 0.03	4.51 ± 0.08^∗^	3.14 ± 0.05^#^
TUNEL (+) per 10^5^ myocytes	45 ± 4	629 ± 91^∗^	316 ± 46^#^
Myocardial infarction size (%)	NA	41.39 ± 5.24^∗^	28.26 ± 4.55^#^

Data were expressed as % or mean ± standard deviation. LVSP: left ventricular systolic pressure; Ea: effective arterial elastance; +dP/dt_max_: maximal rate of pressure increase; Ees: end-systolic elastance; SW: stroke work; −dP/dt_max_: maximal rate of pressure decline; EDP: end-diastolic pressure; EDPVR: end-diastolic pressure-volume relationship; *τ*: isovolumic relaxation time constant; PVA: pressure-volume area; LV: left ventricle; TUNEL: terminal dUTP nick end labeling; NA: not applicable. ^∗^*P* < 0.05 vs the sham-operated group and ^#^*P* < 0.05 vs the untreated heart failure group.

## Data Availability

The data used to support the findings of this study are available from the corresponding author upon request.
